# Anti-inflammation effects of hydrogen saline in LPS activated macrophages and carrageenan induced paw oedema

**DOI:** 10.1186/1476-9255-9-2

**Published:** 2012-02-02

**Authors:** Zheng Xu, Jiangrui Zhou, Jianmei Cai, Zhen Zhu, Xuejun Sun, Chunlei Jiang

**Affiliations:** 1 Department of Nautical Medicine, Second Military Medical University, 800 Xiangyin Road, Shanghai 200433, P.R. of China

**Keywords:** hydrogen saline, macrophages, TNF-α, anti-inflammation, carrageenan

## Abstract

**Background:**

Oxidative stress is thought to play an important role in the pathogenesis of inflammation. Recent studies have found that hydrogen gas has the effect of eliminating free radicals. Whether hydrogen saline (more convenient to be used than hydrogen gas) has the anti-inflammation effect or not is still unknown.

**Methods:**

Carrageenan-induced paw oedema and LPS-activated macrophages are studied in this article. Injection of carrageenan into the foot of a mouse elicited an acute inflammatory response characterized by increase of foot volume and infiltration of neutrophils. While tumor necrosis factorα(TNF-α) secreted by activated macrophages was determined by ELISA and real-time PCR.

**Results:**

All parameters of inflammation (foot volume, infiltration of neutrophils, amount of TNF-α and the level of TNF-α's mRNA) were attenuated by the hydrogen saline treatment.

**Conclusion:**

As a more convenient way than inhaling H_2_, hydrogen saline exhibits a protective effect against inflammation and it might provide a novel therapeutic approach for inflammatory diseases.

## Background

Oxidative stress is thought to play an important role in the pathogenesis of inflammation not only through direct injurious effects, but also by involvement in molecular mechanisms [1]. Among the complicated factors involved in the process of inflammation, reactive oxygen species (ROS) and reactive nitrogen species (RNS), such as the hydroxyl radical (^•^OH), superoxide anion (O_2_^-^), hydrogen dioxide (H_2_O_2_), nitric oxide (NO) and peroxynitrite (ONOO^-^), appear to be critical elements. There is a large amount of evidence showing that the production of reactive species such as O_2_^•-^, H_2_O_2_, and ^•^OH occurs at the site of inflammation and contributes to tissue damage [2,3]. By using inhibitors of NOS, the severity of inflammation was reduced, which demonstrates the role of NO^• ^in the pathogenesis associated with various models of inflammation [4-6]. In addition to NO^•^, ONOO^- ^is also generated during inflammation damage [3,6]. The involvement of ONOO^- ^in these conditions is strongly manifested by direct measurements. There is immunocytochemical documentation (increased nitrotyrosine immunoreactivity in the inflamed tissues) of augmented ONOO^- ^production in many inflammation diseases, such as ileitis [7], endotoxin-induced intestinal inflammation [8] and arthritis[9]. Furthermore, the ability of ONOO^- ^to cause severe colonic inflammation has also been documented [10]. Whereas, detoxification system for ^•^OH and ONOO^- ^in vivo has not been found; therefore, scavenging of ^•^OH and ONOO^- ^turns out to be a vital antioxidant process[11]. It has been reported recently that H_2 _selectively reduced ^•^OH and ONOO^-^[12]. So, as a free radical scavenger, H_2 _may have the effect of anti-inflammation. Recently, molecular hydrogen has been proved effective in curing concanavalin A-induced hepatitis[13] and colon inflammation induced by dextran sodium sulfate[14]. However, inhalation of hydrogen gas may not be convenient for therapeutic use. A brief report has suggested that consumption of water containing hydrogen at a saturated level (hydrogen saline) reduces oxidative stress in rats[15]. Thus, we expect to examine the effects of hydrogen saline on inflammation models.

Macrophages are considered to be an essential participant in inflammation [16]. When activated by endotoxin, macrophages produce inflammatory cytokines, which in turn activate other macrophages and other nearby cells to promote more inflammatory cytokines. Tumor necrosis factor-alpha, as one of these inflammatory cytokines, has a decisive function in the process of inflammation[17], and could represent the severity of inflammation.

In this study, we expect to examine whether the hydrogen saline has the anti-inflammation effect on both animal and cellular inflammation models.

## Methods

### Hydrogen saline

Molecular hydrogen (H_2_) dissolved in saline under high pressure (0.6 MPa) to a supersaturated level for 2 hours[18]. Hydrogen saline was freshly prepared every week. The hydrogen concentration was detected by gas chromatography, the concentration of hydrogen being 0.6 mM in each experiment. Hydrogen saline degassed by gentle stirring was used for control animals.

### Animals and Carrageenan-induced paw oedema

Male BALB/c mice (20-25 g) were housed in a controlled environment and provided with standard rodent chow and water. To test inhibitory effects on acute inflammation in an animal model, paw edema was induced by subcutaneous injection of 0.05 mL of carrageenan (1%) into the right hind paw, as was previously described [19]. Right after the carrageenan administration, the animals received an i.p. injection of either saline(5 ml/kg) or hydrogen saline(5 ml/kg). The paw volume was measured by a volume measuring instrument(YLS-7B from Gene&I) at each hour point after edema induction. The increase in percentage of paw volume was calculated based on the volume difference between the normal and abnormal paws (with or without carrageenan injection). Seven animals per group were tested. The Animal and Ethics Review Committee at the Second Military Medical University evaluated and approved the protocol used in this study.

### Histological examination

After 4 h of carrageenan treatment, mice were sacrificed, and the tissue of the paws was removed and fixed in alfac solution. Each sample was embedded in paraffin wax, sectioned at 5 μm and stained with hematoxylin-eosin. A representative area was selected for qualitative light microscopic analysis of the inflammatory cellular response with a 20× objective [20]. Five slices from each animal were analyzed and a minimum of three animals for each treatment was taken.

### The detection of 3-nitrotyrosine(3 -NT) in the plasma

3-nitrotyrosine(3-NT) is the metabolite of the ONOO^- ^, and it is stable and easier to be detected than ONOO^- ^. What's more, it can reflect the concentration of ONOO^- ^. After 4 h of carrageenan treatment, mice were sacrificed. The blood was collected and centrifuged at 3000 rpm. 3-NT concentration was determined in the cell free supernatant by enzyme-linked immunosorbent assay(ELISA). Antibody-matched pairs and respective standards were purchased from R&D system(Minneapolis, Minn, U.S.A), and were used according to the manufacturer's instructions. The sensitivity for the 3-NT ELISA assay is 2 nmol/L.

### Tissue MPO activity assay

The activity of tissue MPO was assessed 4 h after injection of carrageenan into the mouse's right hind paw. Samples were placed in 0.75 ml of 80 mM phosphate-buffered saline (PBS), pH 5.4, containing 0.5% hexadecyltrimethylammonium bromide, and were then homogenized (45 s at 0°C) in a motor-driven homogenizer. The homogenate was decanted into a microfuge tube, and the vessel was washed with a second 0.75 ml aliquot of hexadecyltrimethylammonium bromide in buffer. The wash was added to the tube, and the 1.5 ml sample was centrifuged at 12,000 × g at 4°C for 15 min. Triplicate 30 μl samples of the resulting supernatant were added to 96-well microtitre plates. For assay, 200 μl of a mixture containing 100 μl of 80 mM PBS, pH 5.4, 85 μl of 0.22 M PBS, pH 5.4, and 15 μl of 0.017% hydrogen peroxide were added to the wells. The reaction was started with the addition of 20 μl 18.4 mM tetramethylbenzidine HCl in dimethylformamide. Plates were incubated at 37°C for 3 min, and then the reaction was stopped by the addition of 30 μl of 1.46 M sodium acetate. pH 3.0. Enzyme activity was determined colorimetrically using a plate reader (ELX800, BioTech Instruments, Inc.) set to measure absorbance at 630 nm and expressed as mOD/mg tissue.

### Isolation of murine peritoneal macrophages

Macrophages were prepared from BALB/c mice as was previously described [21]. Briefly, peritoneal macrophages were harvested from 2 to 3 BALB/c mice, which had been injected intraperitoneally with 1 ml of thioglycollate three days before sterile peritoneal lavage with 5 ml of Hank's balanced salt solution. The collected cells were seeded and cultured in RPMI1640 containing 10% heat-inactivated FBS, 100 IU/ml penicillin and 100 μg/ml streptomycin at a density of 5 × 10^6 ^cells/well. The cells were allowed to adhere for 1 h to a 48-well culture plate at 37°C in a 5% CO_2 _incubator. Then the cultures were washed twice with RPMI1640 to remove nonadherent cells prior to the addition of 400 μl of fresh RPMI1640 containing 10% FBS. The purity of the adherent macrophages was assessed by Giemsa staining(> 95%).

### RT-PCR

Total RNA was isolated from macrophages using TriZol Reagent. Total RNA was reverse-transcribed into cDNA and then PCR amplification of the cDNA was performed. The forward and reverse primer sequences of PCR primers were as follows: murine TNF-α: F 5'-CCC TCA CAC TCA GAT CAT CTT CT-3', R 5'-GCT ACG ACG TGG GCT ACA G-3'; murineβ-actin: F 5'-CTT TGC AGC TCC TTC GTT GC-3', R 5'-ACG ATG GAG GGG AAT ACA GC-3' [22]. An aliquot of the total RNA was reverse-transcribed by RAV2 reverse transcriptase (20 U/ul, TAKARA, Japan) and the oligo-dT primer(300 pmol) in a total volume of 40 ul reaction according to the manufacturer's instructions. cDNAs were analyzed immediately or stored at -20°C until use. Real-time PCR assay was carried out with LightCycler (Roche Diagnostics.Germany) using LightCycler FastStart DNA Master SYBR Green I (Roche Diagnostics). 1 ul of cDNA was added to a 19 ul of reaction mixture including MgCl_2 _at the optimal concentration, 20 pmol of each primer and 2 ul of a LightCycler FastStart DNA Master SYBR Green I. Then the mixture was incubated in LightCycler under the following conditions: at 95°C for 10 min for denaturation, followed by 50 cycles of 94°C for 1 second, each of the optimal annealing temperature for 5 seconds, 72°C for 10 seconds and finally cooling to 40°C[23]. We use the double stranded DNA-binding dye SYBR Green I to estimate the expression level of cytokine mRNA[24]. Transcripts of beta-actin, as a housekeeping gene, were quantified as endogenous RNA of reference to normalize each sample. Relative quantities were estimated by the delta-delta-Ct method. The results were normalized as relative expression in which the average value of the TNF-α mRNA was divided by the average value of beta-actin mRNA.

### Cell culture and detection of TNF-α in the supernatant of the activated macrophages

RAW 264.7 murine macrophages were obtained from the American Type Culture Collection (Manassas, USA). These cells and murine peritoneal macrophages were cultured in a 48-well culture plate with 400 μl RPMI1640 in each well and maintained at sub-confluence in a 95% air and 5% CO_2 _humidified atmosphere at 37°C [25]. The medium used as the routine subculture was RPMI1640 supplemented with 10% fetal bovine serum (FBS), penicillin (100 units/mL) and streptomycin (100 μg/mL). TNF-α concentration was determined in the cell free supernatant obtained after 4 h macrophages co-culture with LPS(100 ng/ml) by enzyme-linked immunosorbent assay(ELISA). Antibody-matched pairs and respective standards were purchased from R&D system(Minneapolis, Minn, U.S.A), and were used according to the manufacturer's instructions. The sensitivity for the TNF ELISA assay is 7 pg/ml.

### Statistics

Statistical comparisons among the groups were assessed by one-way analysis of variance (ANOVA). When F ratios were significant (P < 0.05), LSD 's post hoc tests between two groups were done using SPSS Statistics (SPSS Inc.). P < 0.05 was considered statistically significant.

## Results

### Effects of hydrogen saline in carrageenan-induced paw oedema

**Figure 1:**
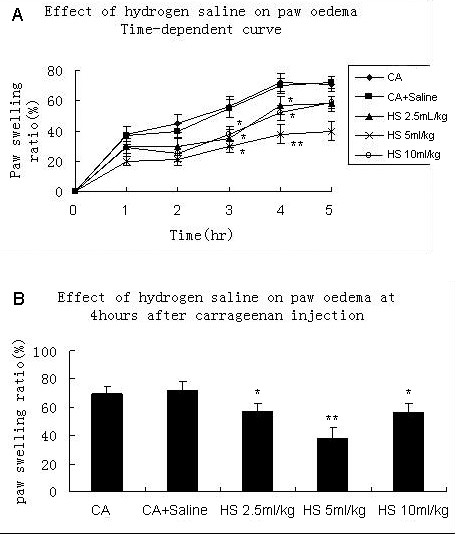
**Effect of treated with different dose of hydrogen saline(range from 2.5 ml/Kg to 10 ml/Kg) on carrageenan-induced paw oedema in mice**. The paw swelling ratio is the percent increase of paw volume. The time-dependent curve has shown us the anti-inflammatory effect of hydrogen saline, and it significantly inhibited the ratio of paw swelling at 4 hours after carrageenan injection. Each value is the mean ± SE (7 mice were tested per group). *P < 0.05 and **P < 0.01. CA = carrageenan treatment. CA + saline = carrageenan and saline treatment. HS = carrageenan and hydrogen saline treatment.

In order to determine the anti-inflammation activity of hydrogen saline in acute-phase inflammation in vivo, a carrageenan-induced paw oedema experiment was conducted. Hydrogen saline showed a remarkable inhibitory activity in carrageenan-induced paw oedema after carrageenan injection. The time-dependent curve shows that the paw swelling ratio will rise till 4 hours after carrageenan injection. **(**Figure 1A) Hydrogen saline showed bell shaped effect of down-regulating carrageenan-induced paw swelling, compared with the vehicle control group, which received an equal volume of vehicle only (saline). (Figure 1A.B). And 5 ml/Kg was the most effective dose, which was similar to the results that our lab had observed(5 ml/Kg was the proper dose for the effects)[18]. It has been reported that the bell shaped phenomenon also exists in the hydrogen gas's effect of anti-ischemia-reperfusion injury[12]. This bell shaped phenomenon is quite hard to explain now and its mechanism needs to be studied in our further experiments.

### Effects of hydrogen saline on histological changes associated with carrageenan-induced mouse paw oedema

**Figure 2:**
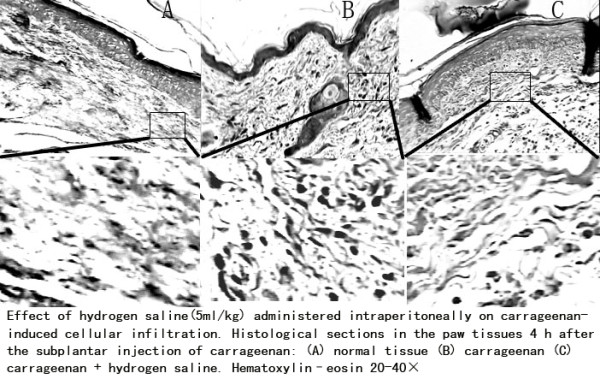
**Effect of hydrogen saline(5 ml/kg) administered intraperitoneally on carrageenan-induced cellular infiltration**. Histological sections in the paw tissues 4 h after the subplantar injection of carrageenan: (A) normal tissue (B) carrageenan (C) carrageenan + hydrogen saline. Hematoxylin-eosin 20-40×, 5 um sections. Five slices from each animal were analyzed and a minimum of three animals for each treatment were taken.

Figure 2 shows some representative photos from the histological changes in the paw tissues following the subplantar injection of carrageenan (0.05 mL/paw) and the anti-inflammation effects of hydrogen saline. The 4 h peak of the carrageenan inflammatory response was found to be associated with subcutaneous oedema along with the heavy infiltration of neutrophils at the site of injection. The treatment of animals with hydrogen saline exhibited a significant decrease in the number of cellular infiltrates.

### Effects of hydrogen saline on the 3-NT in the plasma

**Figure 3:**
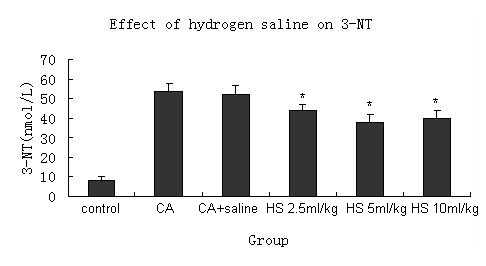
**Hydrogen saline(range from 2.5 ml/kg to 10 ml/kg) decreased 3-nitrotyrosine(3-NT) concentration in the serum**. Serum was collected 4 h after carrageenan treatment. 3-NT was measured with an ELISA(7 mice were tested per group).*P < 0.05 compared with CA+saline group. CA = carrageenan treatment. CA + saline = carrageenan and saline treatment. HS = carrageenan and hydrogen saline treatment.

ONOO^- ^is unstable and can be deoxidized very soon. 3-NT is stable and easy to be detected, and it can reflect the concentration of ONOO^- ^. Figure 3 and Table 1 show that the hydrogen saline can decrease the concentration of 3-NT in the plasma.

**Table 1:** Effect of hydrogen saline on 3-NT and MPO activity

Group	Time after carrageenan injecton	3-NT(mOD/mg tissue)	MPO(nmol/L)
Control	4 hours	8.03 ± 1.96	3.89 ± 1.23

CA	4 hours	53.91 ± 4.11	25.26 ± 2.51

CA+saline	4 hours	52.11 ± 4.93	24.35 ± 3.07

HS 2.5 ml/kg	4 hours	43.88 ± 3.12	20.84 ± 2.17

HS 5 ml/kg	4 hours	37.92 ± 4.21	14.88 ± 1.89

HS 10 ml/kg	4 hours	40.47 ± 3.87	21.92 ± 2.52

Each value represents the mean ± S.E.M. n = 7. *p < 0.05 **P < 0.01 compared with CA + saline group. Student's t-test after analysis of variance. CA = carrageenan treatment. HS = carrageenan + hydrogen saline treatment.

### Effect of the hydrogen saline on MPO activity

**Figure 4:**
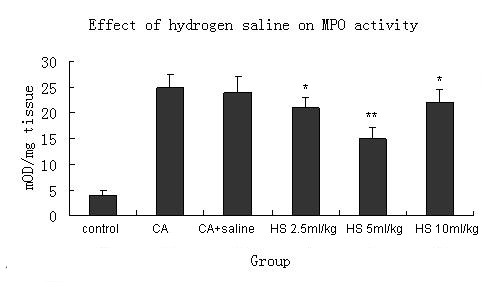
**Effect of different dose of hydrogen saline administered intraperitoneally on myeloperoxidase activity in supernatants of homogenates from carrageenan-treated paws**. Paw myeloperoxidase activities were measured at 4 h after carrageenan treatment. Each bar represents the mean of 7 animals and vertical lines show the S.E.M. *P < 0.05, **P < 0.01. CA = carrageenan treatment. CA + saline = carrageenan and saline treatment. HS = carrageenan and hydrogen saline treatment.

The neutrophil migration into carrageenan-stimulated mouse paws was indirectly determined by means of MPO activity. Treatment with different doses of hydrogen saline significantly prevented the increase in MPO activity induced by carrageenan (Figure 4, Table 1).

### RT-PCR

**Figure 5:**
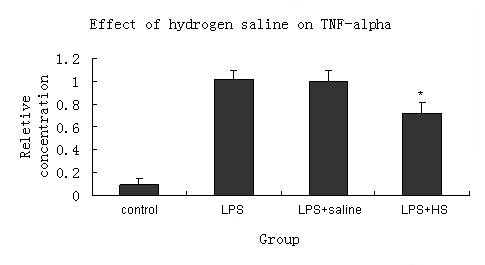
**Expression of mRNA for TNF-α in macrophages cultured with LPS+hydrogen saline(40 μl was added in each well), LPS+saline and LPS**. Cytokine's relative concentration is expressed as means ± SE. *P < 0.05 VS. LPS+saline group. The hydrogen saline had the effect of downregulating TNF-α mRNA. HS = hydrogen saline.

Expression of activated macrophage mRNA was determined 1 hr after the treatment of hydrogen saline or saline. TNF-α mRNA of activated macrophages treated with hydrogen saline was lower than that of those treated with saline.(Figure 5)

### Effects of hydrogen saline on TNF-αproduced by LPS-activated macrophages

**Figure 6:**
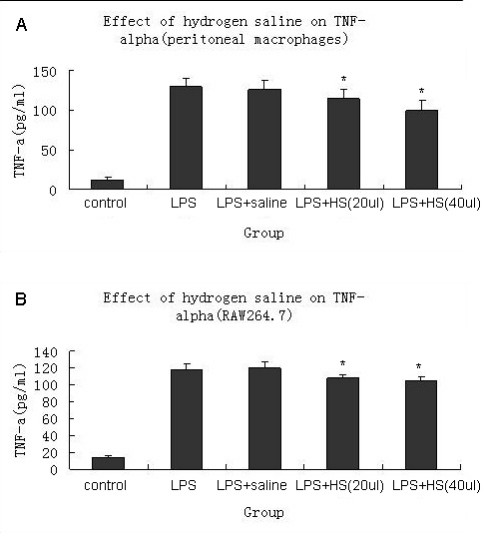
**Hydrogen saline(20 or 40 μl was added in each well) decreased proinflammation cytokine(TNF-α) concentrations in culture supernatants**. Supernatants were collected 4 h after stimulation. TNF-α was measured with an ELISA(7 samples were tested per group).*P < 0.05 compared with LPS+saline group. The murine peritoneal macrophages(A) and the RAW264.7 macrophage cell line(B) had the samilar results. HS = hydrogen saline.

The production of TNF-αfrom activated macrophages which were treated with saline proved much more than that of those treated with hydrogen saline. This result was similar in both murine peritoneal macrophages(Figure 6A) and RAW 264.7 murine macrophages (Figure 6B) indicating that hydrogen saline was able to inhibit the TNF-α production of macrophages.

## Discussion

In this study, hydrogen saline inhibited carrageenan-induced paw oedema, and suppressed the production of TNF-αby activated macrophages. The anti-inflammation effects of hydrogen saline on inflammation models seem related to its function of scavenging radicals and down-regulating TNF-αproduction, because free radicals and TNF-α play a critical role in the process of inflammation. These observations indicate that the hydrogen saline may have clinical potentials for the treatment of inflammation diseases.

The amount of hydrogen dissolved in saline is limited but it does have the effect of anti-inflammation. Our concentration used in the experiment has proved to be effective in curing other kinds of oxidant-stress related injury such as atherosclerosis[26] and type 2 diabetes[27]. Other scientists found that much lower concentration of hydrogen still has its effect[28]. So, the limited amount of hydrogen should be sufficient for exhibiting the anti-inflammation effect. Hydrogen may reach tissues by vessel conveying. The hydrogen is a liposoluble small molecular which can easily penetrate cell membrane, so it can be absorbed by mesenteric vessels and conveyed to any part of the body.

We found the anti-inflammation effects of hydrogen saline in a vivo inflammation model. Reportedly, carrageenan-induced paw edema is a highly sensitive tool to evaluate the efficacy of acute inflammation [19,29]. To decrease carrageenan-induced paw swelling by reducing levels of IL-8, IL-1β, TNF-α, NO via iNOS inhibition, and PGE2 via COX-2 inhibition, have all been documented [30,31]. In the present study, hydrogen saline showed dose-dependent inhibitory activity in carrageenan-induced paw edema.

The reason why we studied TNF-α secreted by macrophages is that, as an important inflammation mediator, TNF-α can reflect the degree of inflammation. Through the measurement of TNF-α we can come to the conclusion that the hydrogen saline has the anti-inflammation effect in cellular inflammation models. Furthermore, as mentioned above, TNF-αplays an important role in the process of inflammation[16,17]. Reducing the level of TNF-αhas the effect of anti-inflammation in the animal model[32]. Therefore, the ability of hydrogen saline to suppress TNF-α production may be one of the factors that exert the anti-inflammation effect in the carrageenan induced paw oedema model.

Inflammation and free radicals have the relationship of mutual promotion. The inflammation can cause the increase of free radicals. Meanwhile, free radicals such as the hydroxyl radicals(^•^OH) and peroxynitrogen (ONOO^-^) play an important role in the progress of inflammation, and inhibiting these free radicals can reduce the severity of inflammation. H_2 _molecule has a very strong covalent bond, so it can not easily react with all the oxidants. However, in recent studies, Ohsawa et al has found that molecular hydrogen can specifically reduce ^•^OH and ONOO^- ^in vitro and induce a therapeutic antioxidant effect in an animal model[12]. The ONOO^- ^and ^•^OH are the strongest oxidants and react indiscriminately with nucleic acids, lipids and proteins resulting in DNA fragment, lipid peroxidation, and inactivation of protein. O_2_^- ^and H_2_O_2 _are detoxified by antioxidant defense enzymes, superoxide dismutase, and peroxidase or glutathione-peroxidase, respectively; however, no enzyme detoxifies ^•^OH and ONOO^-^. Therefore, the ability of hydrogen saline to reduce or eliminate ^•^OH and ONOO^- ^may be responsible for the anti-inflammation effect observed in this study. Our study suggests that hydrogen saline has the effects of anti-inflammation and scavenging peroxynitrogen. As we know, hydrogen saline's direct and main effect is eliminating free radicals. So our results suggest that the anti- inflammation effect of hydrogen saline may be caused by eliminating the free radicals.

Our study suggests that the effect of hydrogen at high concentration still has the anti-inflammation effect, though not as effective as 5 ml/Kg. This bell shaped effect phenomenon is quite strange and its mechanism is not clear. The experiments of Ikuroh Ohsawa[12] also has the similar results, but it does not give the reason. We think that the bell shaped effect of 3-NT and MPO may play a role in the hydrogen's bell shaped effect of anti-inflammation. Furthermore, the negative feedback system may also play a role in the down-regulating anti-inflammation effect of hydrogen. Meanwhile, the high concentration of hydrogen may affect the free radicals which are involved in the normal signal transduction system, and this may also explain the poor effect of anti-inflammation of hydrogen at high concentration. All the possible mechanisms are hypotheses which need to be proved in our future study.

Above all, we found that the hydrogen saline had the effect of anti-inflammation and the mechanism may work by reducing ONOO^-^. Furthermore, the down-regulated TNF-α production by macrophages may also play a role in the anti-inflammation effect. Although further investigations are still required to clarify the detailed mechanism, we could conclude that hydrogen saline is a potential candidate for the therapy of inflammatory diseases, which is more convenient to administer than inhaling hydrogen.

## Conclusion

We found that the hydrogen saline has the effect of anti-inflammation to the carrageenan induced paw oedema and LPS-activated macrophages. Our results clearly demonstrate that hydrogen saline treatment exerts a protective effect and offers a novel therapeutic approach for the treatment of inflammation diseases.

## Competing interests

The authors declare that they have no competing interests.

## Authors' contributions

ZX carried out the animal experiment and drafted the manuscript. JZ carried out the immunoassays and real-time PCR test. JC and ZZ participated in the MPO and 3-NT test. XS participated in the design of the study and performed the statistical analysis. CJ conceived of the study, and participated in its design and coordination. All authors read and approved the final manuscript.
